# Disruption Events in the HPV18 *E1* and *E2* Genes in Precancerous Cervical Lesions

**DOI:** 10.3390/ijms26146974

**Published:** 2025-07-20

**Authors:** Eirini Agnanti, Dimitris Tsakogiannis, Theologos Papadopoulos, Konstantinos I. Arvanitidis, Zaharoula Kyriakopoulou, Ioannis Karakasiliotis, Christine Kottaridi

**Affiliations:** 1 Laboratory of General Microbiology, Department of Genetics, Development and Molecular Biology, School of Biology, Aristotle University of Thessaloniki, 54124 Thessaloniki, Greece; agirini2@gmail.com (E.A.); theologos.pap@gmail.com (T.P.); 2 Research Center, Hellenic Anticancer Institute, 10680 Athens, Greece; 3 Laboratory of Pharmacology, Department of Medicine, Democritus University of Thrace, 68100 Alexandroupolis, Greece; karvanit@med.duth.gr; 4 Dimokritos Diagnostic Laboratories, 68100 Alexandroupolis, Greece; 5 Department of Environmental Sciences, School of Technology, University of Thessaly, Gaiopolis Campus, 41500 Larissa, Greece; zahkyr@uth.gr; 6 Laboratory of Biology, Department of Medicine, Democritus University of Thrace, 68100 Alexandroupolis, Greece; ioakarak@med.duth.gr

**Keywords:** HPV18, integration, *E1*, *E2*

## Abstract

Human papillomavirus 18 (HPV18) is the second most oncogenic high-risk HPV genotype, after HPV16, and is responsible for about 15% of cervical cancer cases worldwide. The integration of high-risk HPV DNA into the host genome leads to the disruption of the *E1* and/or *E2* genes, which is considered a risk factor for viral-induced carcinogenesis. This study examined the disruption events of HPV18 *E1* and *E2* genes in precancerous cervical lesions to investigate the rates and sites of gene disruption in the Greek population. The complete *E1* and *E2* genes were amplified using three and four overlapping primer sets, respectively. Extensive analysis revealed that the disruption/deletion events of the *E1* and *E2* genes were detected in all grades of cytology-determined lesions, with high frequency. *E2* gene disruption was significantly related to LSIL+ cases (Fisher’s exact test, *p* = 0.022). No significant association was found in the analysis of the *E1* gene. Additionally, no preferential sites of *E1*/*E2* gene disruption were detected. This is the first study to provide evidence of disruption events of the HPV18 *E1* gene. The data from the current analysis suggest that disruption of the *E2* gene could be a significant marker for the progression of cytology-determined cervical dysplasia. However, future studies are required to evaluate whether different geographic populations have particular profiles regarding the rates and sites of gene disruption to further determine population-specific biomarkers.

## 1. Introduction

Human Papillomaviruses (HPVs) are small, non-enveloped, double-stranded DNA viruses approximately 8000 bp in length that infect the basal layer of mucosal and cutaneous epithelia in a broad spectrum of higher vertebrates [1,2]. They are members of the Papillomaviridae family, which consists of 53 genera, five of which infect humans (Alphapapillomaviruses, Betapapillomaviruses, Gammapapillomaviruses, Mupapillomaviruses, Nupapillomaviruses [3,4]. The genome of HPVs is classified into three regions: (i) the early region that encodes the early genes *E1, E2, E4, E5, E6,* and *E7*; (ii) the late region that encodes the late genes *L1* and *L2*; and (iii) the long control region (LCR) [5,6]. The classification of HPVs in different genera is based on sequence similarity of the *L1* gene. Different genera have less than 60% *L1* sequence similarity, whereas species within a genus have 60–70% *L1* sequence homology [7,8]. Moreover, different HPV genotypes share less than 90% sequence similarity within the *L1* gene [7,8]. According to their tumorigenic capacity, mucosal Alphapapillomaviruses are further classified into high-risk (HR-HPV) and low-risk (LR-HPV) genotypes [3,4]. Persistent infection with HR-HPV genotypes is the main cause of severe dysplasia and cervical cancer development [9].

Cervical cancer is the fourth most common type of cancer among women, globally. In 2022, about 660,000 new cases were diagnosed and approximately 350,000 women died from the disease [10]. HPV16 is the most prevalent genotype detected in more than 60% of cervical cancer cases, followed by HPV18 [8]. In 2023, the estimated prevalence of HPV16/18 among women with cervical cancer was 74% in Europe, 68.9% in Asia, 68.2% in America, and 67.2% in Africa [10]. However, the distribution of HPV16/18 in cervical tumors varies among different countries in Europe. For example, Croatia had 82.9%, Bulgaria 80.3%, France 75.6%, Italy 72.2%, Spain 63.1%, and Greece 52.3% [11]. In 2025, the overall prevalence of HPV16 and HPV18 in Greek women was 1.8% and 0.7%, respectively [12]. While HR-HPV infection is the leading cause of cervical cancer development, it is also implicated in the pathogenesis of several other types of cancer, including oropharynx, penis, vulva, anus, and vaginal cancer [13].

HPVs have developed various mechanisms to promote their genome replication. These molecular mechanisms involve preventing cell-cycle arrest, cellular senescence, and apoptosis, which can lead to severe genomic instability and cancer growth [14,15]. The function of viral E6 and E7 oncoproteins plays a pivotal role in HPV-induced carcinogenesis. The viral oncoproteins are implicated in uncontrolled cell proliferation, as they inactivate the two major tumor suppressor proteins p53 and pRB, respectively [16,17]. Specifically, the E6 oncoprotein forms a complex with the E3 ubiquitin ligase E6-associated protein (E6AP). Subsequently, the E6/E6-AP complex binds to p53, leading to ubiquitin-mediated proteolysis [16]. On the other hand, the E7 oncoprotein targets pRB and releases the E2F factor from the pRB/E2F suppressor complex, compelling the infected cells to enter the S-phase prematurely [18,19].

An additional molecular mechanism involved in cervical carcinogenesis is the integration of viral DNA into the host chromosome. As the disease progresses, the viral DNA becomes integrated into the host chromosome, leading to genomic structural damage at the site of viral insertion [20,21]. Upon integration, the circular viral DNA is linearized through the disruption of *E2* and/or *E1* genes, while the LCR and *E6*, *E7* oncogenes remain intact and integrated into the host chromosome [6,22,23,24]. Despite chromosomal aberrations, the integrant-derived E6, E7 transcripts are more stable than those of episomal derived transcripts, leading to elevated production of the E6, E7 oncoproteins [25]. This phenomenon gives infected cells a selective growth advantage, allowing for tumor development. The viral DNA can be found in three physical forms: a circular episomal form, a linearized integrated form, and a mixed form (episomal and integrated) [26,27].

The detection of integrated viral genomes has emerged as a valuable biomarker for the development of cervical cancer [28]. Viral integration often occurs at the early stages of the HPV life cycle and its frequency increases with the severity of cervical dysplasia [22]. Moreover, studies have shown that the integration frequency varies among different HR-HPV genotypes [29,30,31]. The integration rate of HPV18 DNA is higher in cases of severe dysplasia and invasive cancer compared to that of HPV16 DNA [29,30,31,32]. Therefore, the presence of an integrated HPV18 genome has been recommended as a significant marker for triaging HPV18 infections with varying clinical effects [26,27,28,29]. Although most studies have focused on the extensive analysis of disruption/deletion events of HPV16 *E1* and *E2* genes and the integration status of HPV16 DNA, little is known about HPV18. Additionally, information about HPV18 *E1* and *E2* gene disruption/deletion events in the Greek population remains limited. This study aimed to analyze the disruption profile of HPV18 *E1* and *E2* genes in HPV18-positive Greek patients to investigate the rates and sites of gene disruption in the Greek population.

## 2. Results

**Figure 1:**
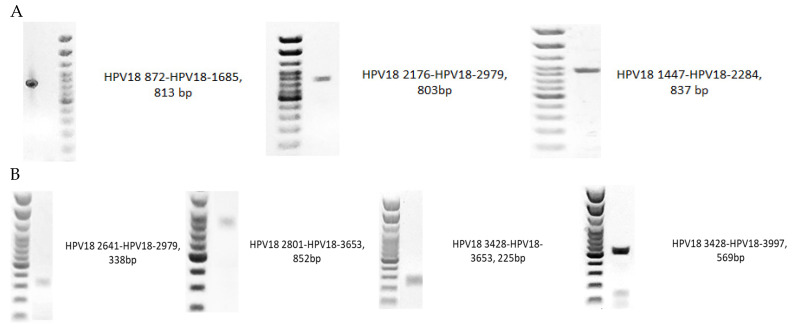
The PCR amplification of genomic regions of the reference Human Papillomavirus 18: (**A**) *E1* gene PCR products; (**B**) *E2* gene PCR products. A 100 bp (base pair) DNA ladder, used as a molecular size marker in gel electrophoresis.

All primers designed for the present study (Table 1) gave a positive result after the amplification of reference HPV18 DNA (Figure 1).

**Figure 2:**
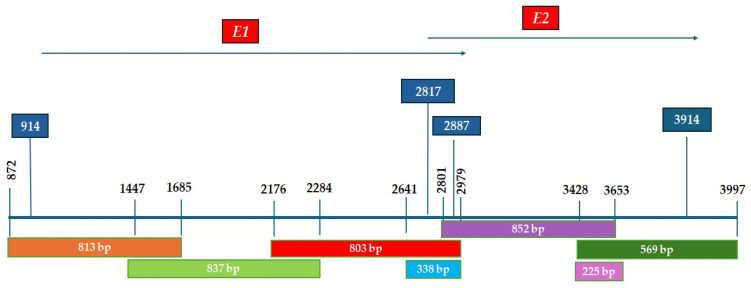
A diagram of the overlapping primer pairs used in the present study, with their nucleotide positions along the *E1* and *E2* genes (Human Papillomavirus 18, NC_001357.1).

Disruption events of HPV18 *E1* and *E2* genes were detected using primer sets that allowed for the amplification of partial overlapping fragments of the *E1* and *E2* genes of viral DNA (Figure 2). Disruption events were determined only for samples that did not show an amplification signal in gel electrophoresis after PCR with one of the newly designed primer sets for *E1* and *E2* genes but were positive for the amplification of HPV18 *E6* and *E7* genes.

**Figure 3:**
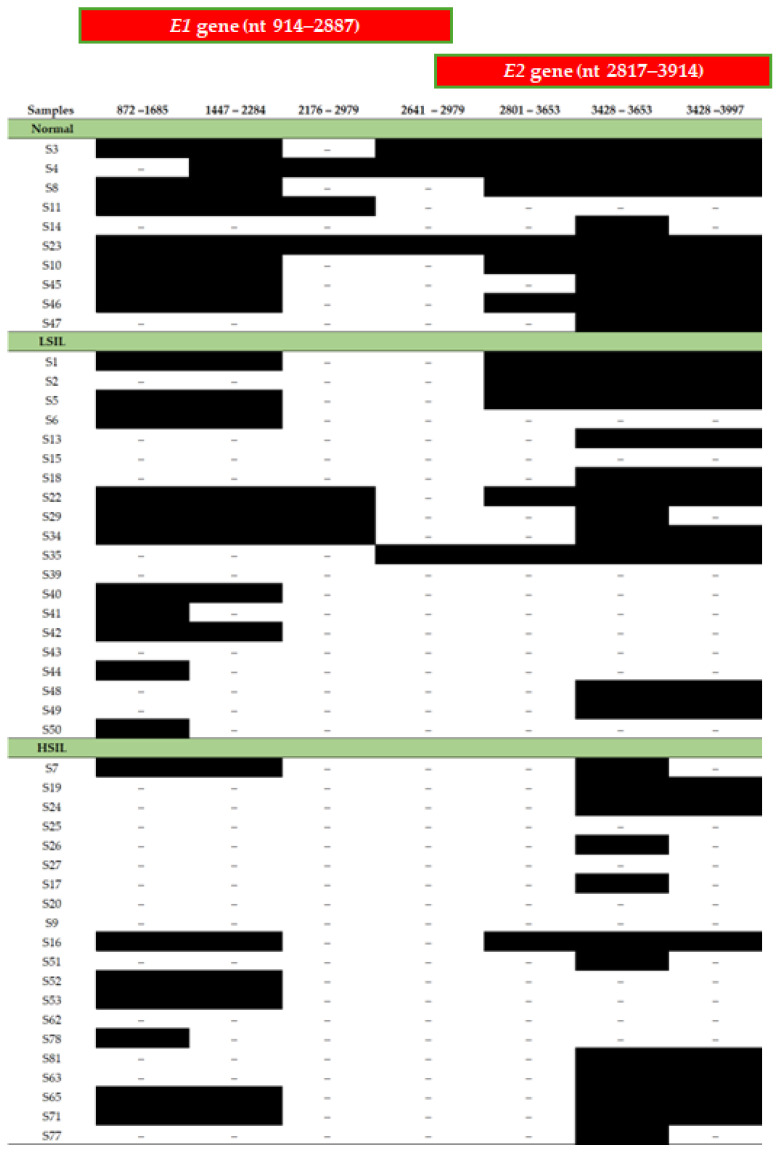
Disruption sites of the HPV18 *E1* and *E2* gene among normal, LSIL, and HSIL samples based on the overlapping primer pairs.

**Figure 4:**
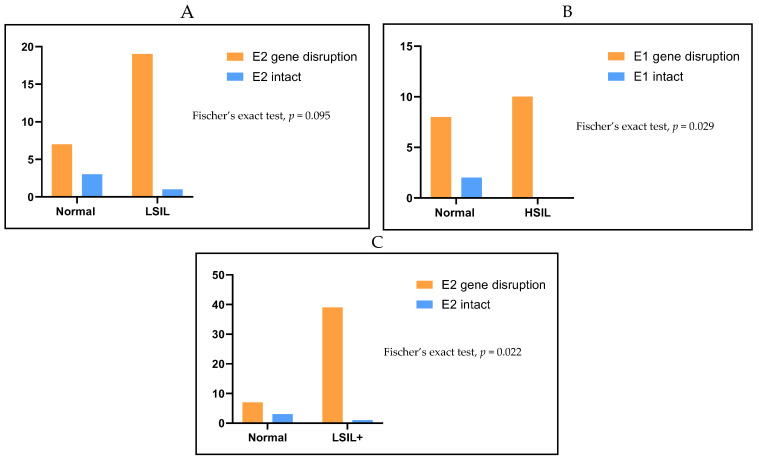
(**A**) Association of *E2* gene disruption between normal and cytology-determined LSIL samples. (**B**) Association of *E2* gene disruption between normal and cytology-determined HSIL cases. (**C**) Correlation of *E2* gene disruption between normal and LSIL+ samples (LSIL and HSIL). The x-axis denotes the different cytological classification groups used in the study, while the y-axis indicates the number of samples corresponding to each category.

All cervical samples examined in this analysis were positive for the amplification of the *E6* and *E7* genes, indicating the integrity of viral DNA. The disruption frequency of the HPV18 *E2* gene was high and was observed in a total of thirty cervical samples (46/50, 92%), (Table 2, Figure 3). *E2* disruption was found in specimens of all stages of cytology-determined cervical dysplasia even among samples with normal cytology results. Specifically, seven out of the ten normal cervical samples (7/10, 70%) showed disruption within the *E2* gene, while three normal cervical cases (3/10, 30%) contained the complete *E2* gene (Table 2, Figure 3). Additionally, the *E2* gene was disrupted in nineteen out of twenty LSIL samples (19/20, 95%), with only one LSIL specimen harboring the complete *E2* gene (1/20, 5%) (Table 2, Figure 3). Finally, the *E2* gene was disrupted in all HSIL samples (20/20, 100%) (Table 2, Figure 3). Statistical analysis revealed that the disruption of the *E2* gene was not associated with LSIL samples when compared to normal cases (Fisher’s exact test, *p* = 0.095), (Figure 4A). However, *E2* gene disruption was significantly related to HSIL samples when compared to normal samples (Fisher’s exact test, *p* = 0.029), (Figure 4B). Furthermore, when LSIL and HSIL cases were grouped together, it was revealed that *E2* gene disruption is significantly related to LSIL+ samples (Fisher’s exact test, *p* = 0.022), (Figure 4C).

The *E2* gene-mapping analysis revealed that disruptions occur at equal frequencies in all positions of the viral gene. Specifically, disruptions were identified from nucleotides 2641 to 2979 in seven normal, nineteen LSIL, and twenty HSIL samples (Figure 3). Disruptions in the *E2* gene from nucleotides 2801 to 3653 were observed in four normal, fifteen LSIL, and nineteen HSIL cases (Figure 3). Additionally, disruptions between nucleotides 3428 to 3653 were found in one normal, nine LSIL, and eight HSIL samples, while disruptions between nucleotides 3428 to 3997 were detected in two normal, ten LSIL, and thirteen HSIL samples (Figure 3).

**Figure 5:**
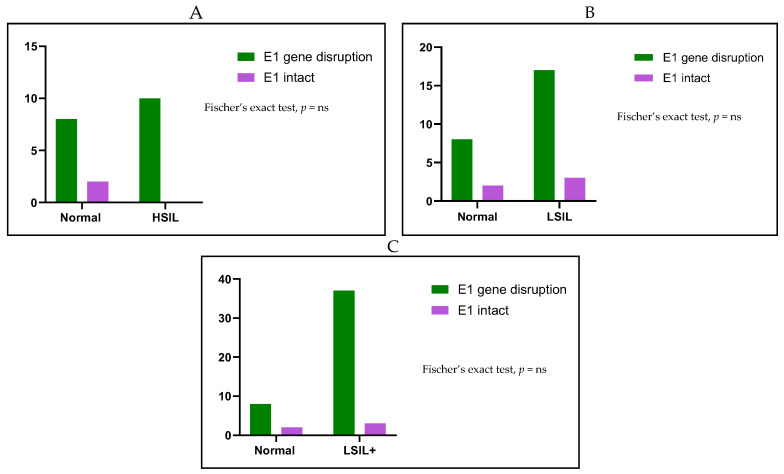
(**A**) Association of *E1* gene disruption between normal and cytology-determined LSIL samples. (**B**) Correlation of *E1* gene disruption between normal and cytology-determined HSIL cases. (**C**) Association of *E1* gene disruption between normal and LSIL+ samples (LSIL and HSIL). The x-axis denotes the different cytological classification groups used in the study, while the y-axis indicates the number of samples corresponding to each category. ns means not significant.

When examining the HPV18 *E1* gene, it was found that disruption events occur in all stages of cytology-determined cervical lesions as well as in samples with normal cytology findings. *E1* gene disruption was identified in a total of forty-five (45/50, 70%) cervical samples (Table 2, Figure 3). Specifically, eight out of the ten normal samples contained disruption events within the *E1* gene (8/10, 80%), while two normal cases had the complete *E1* gene (2/10, 20%), (Table 2). Additionally, the *E1* gene was disrupted in seventeen out of the twenty LSIL cases (17/20, 85%), whereas only three LSIL samples had the complete *E1* gene (3/20, 15%), (Table 2, Figure 3). Similarly, *E1* gene disruption was present in all HSIL samples (20/20, 100%). There was no statistically significant association between gene disruption and the grade of cervical dysplasia, as a high *E1* gene disruption rate was observed in normal, LSIL, and HSIL cases (Fisher’s exact test, *p* = ns), (Figure 5).

The outcomes of the *E1* gene-mapping analysis revealed that there is no preferential site of gene disruption. Specifically, disruptions from nucleotides 872 to 1685 were found in three normal, nine LSIL, and thirteen HSIL samples (Figure 3). Additionally, disruptions from nucleotides 1447 to 2285 were detected in two normal, twelve LSIL, and fourteen HSIL cases, while disruptions form nucleotides 2176 to 2979 were recorded in seven normal, fourteen LSIL, and twenty HSIL samples (Figure 3). Combining the outcomes of the mapping analysis of the HPV18 *E1* and *E2* genes, it was concluded that a disruption pattern involving both *E1* and *E2* genes is present in the majority of samples. This pattern was detected in seven normal, sixteen LSIL, and all HSIL cases.

## 3. Discussion

The integration of HR-HPV DNA into the host chromosome is a key feature of viral infection and a determining factor for cervical cancer development [22,33]. Interestingly, more than 80% of HPV-positive cervical tumors contain the integrated viral form [30,34]. Previous analyses have implied that all HR-HPV genotypes show evidence of integration events in cancer cases, while LR-HPV genotypes are rarely integrated [35,36,37]. The frequency of viral integration varies among different HR-HPV genotypes [29]. Specifically, it has been suggested that over 80% of HPV18- and HPV45-positive cervical cancers harbor the integrated form of viral DNA, while 50–70% of HPV16-positive cervical tumors contain the integrated viral form [21,38]. The integration of viral DNA into the host chromosome causes the disruption of the *E1* and *E2* genes [22]. In this study, we investigated the disruption events of the HPV18 *E1* and *E2* genes in cytology-determined precancerous cervical cases from Greek women, to assess the frequencies and sites of the disruption of HPV18. Gene disruption sites were identified in specimens that tested negative for at least one amplified fragment. The amplification of the *E6* and *E7* genes was used as an internal control to evaluate the integrity of viral DNA. Samples containing the complete *E1* and *E2* genes were considered to have episomal or mixed DNA forms, while disruptions in these genes indicated the integration of HPV18 DNA into the host genome.

Previous analyses suggested that HPV18 integration alone is not capable of leading to the development of severe lesions and cancer, as high rates of viral integration were observed even at the early stages of cervical dysplasia [32,39,40]. In the present analysis, the disruption/deletion of HPV18 *E1* and *E2* genes was detected in all grades of cervical dysplasia with high frequency. Therefore, it was anticipated that viral gene disruption and subsequently HPV18 DNA integration might be common and early-stage events in viral infection [32,39,40]. Moreover, our findings indicate that disruption/deletion of the HPV18 *E2* gene is a frequent event even among samples with normal cytology. Disruption events in the HPV18 *E2* gene have been previously detected in normal cases, supporting the assumption that the integration of the HPV18 genome occurs early in the progression of cervical disease [32,40]. However, further studies are required to confirm this hypothesis. Furthermore, our results indicate that *E2* gene disruption was significantly related to LSIL+ cases (Fisher’s exact test, *p* = 0.022). As a result, it was concluded that an extensive mapping analysis of the *E2* gene could serve as a significant marker providing valuable information about the progression of cervical disease. However, more analyses in precancerous lesions are required to support this observation. On the other hand, no significant association was found considering *E1* gene analysis, as high rates of *E1* gene disruption were detected in normal, LSIL, and HSIL samples. This is the first study to provide evidence of the disruption of the HPV18 *E1* gene in normal samples and precancerous lesions.

Another aspect of the present study was to investigate whether there are specific disruption motifs within the *E1* and *E2* genes. Previous findings regarding the analysis of disruption events in HPV16 *E1*/*E2* genes revealed a specific disruption pattern associated with the severity of cervical malignancy. Specifically, it was found that HPV16 *E2* gene disruption exclusively occurs in HSILs and cervical cancer cases, while HPV16 *E1* gene disruption is detected in all grades of lesions and cervical cancer samples [23,24,41]. Little is known about the disruption pattern of *E1* and *E2* genes in precancerous lesions and cervical cancer incidences for HPV18. A previous analysis conducted in invasive cancer cases showed that disruption of *E1* and *E2* was more frequently detected in HPV18 (84%) than HPV16 (30%) [42]. Moreover, it was suggested that *E2* gene disruption was most common, followed by disruption of both genes, while single disruption events of the *E1* gene were rare for HPV18 [42]. In the present analysis, a disruption pattern involving both *E1* and *E2* genes was detected in the majority of samples in all grades of cytology-determined cervical dysplasia. Additionally, no preferential sites of *E1* and *E2* gene disruption were detected in HPV18 DNA in the current analysis. These results contradict previous findings in invasive cervical cancer cases, which reported that the most frequently disrupted region of the *E2* gene was between nucleotides 3369 and 3739, with no preferential sites for *E1* gene disruption [42]. Further analyses in different populations and different grades of lesions are required to assess whether there are differences in the disruption motifs and preferential sites of gene disruptions in HPV18 among various geographic populations.

In conclusion, this study is the first to describe disruption events of HPV18 DNA in precancerous lesions in the Greek population. While we fully acknowledge the limitations posed by the small patient cohort, we believe that the study offers valuable preliminary evidence on the involvement of HPV18 integration in cervical carcinogenesis. Results from the present analysis suggest that the disruption of both genes could be a common and early event during the HPV18 life cycle, while no preferential sites of *E1* and *E2* gene disruption were detected. The analysis of the HPV18 *E1* and *E2* genes and, specifically, the extensive mapping analysis of the *E2* gene, could serve as a significant marker that could provide evidence about the progression of cytology-determined cervical malignancy. However, more studies are required to evaluate whether different populations have particular profile of rates and sites of gene disruption in order to determine population-specific markers in the future. It should also be noted that cytology is not the gold standard for disease grading, and as such, our findings—particularly those concerning cases classified as low-grade or no disease—should be interpreted with caution given this limitation.

## 4. Materials and Methods

### 4.1. Cervical Samples

Fifty cervical samples positive for HPV18 were selected for inclusion in the study. We included non-pregnant Greek women, 25–64 years of age. All specimens were prepared using Liquid-Based Cytology (LBC) and processed according to standard cytopathological protocols. The samples were collected using a cytobrush and immediately transferred into a vial of PreservCyt transport medium. Following collection, samples were stored at 4 °C until they were further processed using molecular biology techniques. The present analysis did not represent a normally screened population, as enrolled patients attended a diagnostic laboratory (Demokritos Diagnostic Laboratory, Efstathiou 1, 68100, Alexandroupoli). Specifically, twenty samples were diagnosed as low-grade squamous intraepithelial lesions (LSIL), twenty samples were characterized as high-grade squamous intraepithelial lesions (HSIL), while ten HPV18 positive cervical samples with normal cytology were included as well. All patients signed an informed consent form and the study was approved by the research committee of the Ethics Committee of the Democritus University of Thrace (approval number: 01/11/2024-ΔΠΘ/ΕHΔΕ/17283/155).

### 4.2. DNA Isolation and HPV Genotyping

ThinPrep samples were centrifuged at 16,000× *g* for 3 min and the pellet was resuspended in 200 μL of 1x PBS. The total DNA was extracted using the chaotropic agent guanidine thiocyanate (GuSCN) [43]. The cervical samples were screened for the presence of HPV18 DNA with an in-house protocol, targeting the HPV18 *E6* gene specifically, as previously described [44].

### 4.3. Amplification of HPV18 E6 and E7 Genes

The HPV18 E6 and E7 genes were amplified to evaluate the integrity of viral DNA using the primer set HPV18-31 5′-AAAAGGGAGTAACCGAAAACG-3′ and HPV18-888 5′-CACGGACACACAAAGGACAG-3′ (Table 1). The primers were designed using the Primer3 program (https://www.primer3plus.com/index.html, accessed on 19 July 2023) and the numbering of nucleotide positions was based on the nucleotide sequence of the prototype HPV18 strain (GenBank ID: NC_001357). The primer set was designed to amplify the complete *E6* gene and a partial fragment of the *E7* gene. Specifically, the forward primer targets 74 bp upstream from the transcriptional start site of the HPV18 *E6* gene, while the reverse primer targets 19 bp upstream from the 3′ end of the HPV18 *E7* gene.

PCR was performed in a final volume of 25 μL. The PCR mixture consisted of 0.4 μΜ of each primer and 2X Dream Taq PCR Master Mix (Thermo Scientific, Waltham, MA, USA). The cycling conditions were as follows: 35 cycles of 30 s at 95 °C, 30 s at 53 °C, and 30 s at 72 °C. The first cycle was preceded by a 2 min denaturation step at 95 °C, and the last cycle was followed by a 10 min elongation step at 72 °C. Amplicons were monitored in a 2% agarose gel stained with 1 μg/mL of ethidium bromide in Tris-borate-EDTA buffer using a 100 bp DNA ladder as a molecular weight marker (Invitrogen, Life Technologies, Paisley, UK).

### 4.4. Primer Validation

All primer sets were designed to match conserved regions among the reference sequence of HPV18 genome and HPV18 variants, identified through multiple sequence alignment. We aligned each primer set with the HPV18 reference genome as well as with sequences from its variant lineages and sublineages. This approach ensured that the primers perfectly matched conserved regions across all variants, minimizing the risk of mismatches that could cause false-negative results. Thus, the primer design accounts for sequence variability within HPV18 to allow for broad and reliable detection. The accession numbers of variants belonging to the A1, A2, A3, A4, A5, A6, A7, A8, B1, B2, B3, and C lineages are listed below. A1 sublineage: EF202143-EF202145; A2 sublineage: EF202146; A3 sublineage: EF202147-EF202149; A4 sublineage: EF202150-EF202151; A5 sublineage: GQ180787; A6 sublineage: KY457833-KY457836; A7 sublineage: KY457837-KY457840; A8 sublineage: KY457826-KY457827; B1 sublineage: EF202153-EF202155; B2 sublineage: KC470224-KC470225; B3 sublineage: EF202152; and C lineage: KC470229-KC470230.

The reference clone of HPV18 was requested from the International Human Papillomavirus Center (https://www.hpvcenter.se/, accessed on15 September 2023), which was distributed to the General Microbiology Laboratory, School of Biology, AUTH after the signing of a proper MTA. The obtained plasmid vector with the reference HPV18 clone was used for the transformation of competent cells and the recombinant viral DNA was extracted. All primers designed in the present study for the amplification of *E1* and *E2* genes were tested using the reference HPV18 DNA.

### 4.5. PCR Amplification of HPV18 Ε1 Gene and HPV18 Ε2 Gene

The complete *E1* gene was amplified using three overlapping primer sets in separate reactions to locate sites of disruptions within the *E1* gene. The primer sets were designed in the present study (Table 1). Each PCR assay was carried out in a final volume of 25 μL with 0.4 μΜ of each primer and 2X Dream Taq PCR Master Mix (Thermo Scientific, Waltham, MA, USA). The cycling conditions were as follows: 35 cycles of 30 s at 95 °C, 30 s at 54 °C (for primer set HPV18-872/HPV18-1685), 30 s at 49 °C (for primer set HPV18-1447/HPV18-2284), 30 s at 50 °C (for primer set HPV18-2176/HPV18-2979), and 30 s at 72 °C. The complete *E2* gene was amplified using four overlapping primer sets in separate reactions, to identify sites of disruption within the *E2* gene. The primers were designed in the current study (Table 1). Each PCR assay was performed in a final volume of 25 μL. Each PCR mixture 0.4μΜ of each primer, 2X Dream Taq PCR Master Mix (Thermo Scientific, Waltham, MA, USA). The cycling conditions were as follows: 35 cycles of 30 s at 95 °C, 30 s at 50 °C and 30 s at 72 °C. In all PCR assays, the first cycle was preceded by a 2 min denaturation step at 95 °C and the last cycle was followed by a 10 min elongation step at 72 °C. The *E1* and *E2* amplicons were electrophoresed on 2% agarose gel.

### 4.6. Statistical Analysis

The relationship between the sites of *E1* and *E2* gene disruptions and the grade of cervical lesions was determined using Fisher’s exact test with GraphPad Prism 6 (GraphPad software, La Jolla, CA, USA). *p*-values were considered statistically significant at the 0.05 cut-off level.

## Figures and Tables

**Table 1:** Primer list. The PCR products were visualized in the absence of disruption events. All primers were designed in the present study. The numbers in the primer names indicate their corresponding positions in the reference HPV18 strain genome (GenBank ID: NC_001357).

HPV18	Primers	Tm (°C)	PCR Product Length (bp)	Position (nt)	Sequence (5′–3′)
*E6* and *E7*	HPV18-31	57	857	31	AAAAGGGAGTAACCGAAAACG
	HPV18-888	60		888	CACGGACACACAAAGGACAG
*E1*	HPV18-872	60	813	872	GTCCTTTGTGTGTCCGTGGT
	HPV18-1685	58		1685	TCCTTCTGCTATTGTTGGGTTT
	HPV18-1447	62	837	1447	GCAGTGTAGACGGTACAAGTGA
	HPV18-2284	56		2284	TGTTGGTATCGCAGGAATTG
	HPV18-2176	54	803	2176	AGCCCAAAAACGACAAATGA
	HPV18-2979	56		2979	TGCCATGTTCCCTTGCTG
	HPV18-2641	58	338	2641	TCCAGCAAAGGATAATAGATGG
	HPV18-2979	56		2979	TGCCATGTTCCCTTGCTG
*E2*	HPV18-2801	57	852	2801	GCACGAGGAAGAGAAGATG
	HPV18-3653	56		3653	CGTCTTTTGTTGTTGCCTGT
	HPV18-3428	60	225	3428	TGTGCAGTACCAGTGACGAC
	HPV18-3653	56		3653	CGTCTTTTGTTGTTGCCTGT
	HPV18-3428	60	569	3428	TGTGCAGTACCAGTGACGAC
	HPV18-3997	60		3997	GGACATGGCAGCACACATAC

**Table 2:** Rates of *E1* and *E2* gene disruption among normal, LSIL, and HSIL.

	***E1* Gene Disruption**	***E1* Intact**	**Total**
	***n* (%)**	***n* (%)**	***n* (%)**
Normal	8 (80)	2 (20)	10 (100)
LSIL	17 (85)	3 (15)	20 (100)
HSIL	20 (100)	0 (0)	10 (100)
Total	45 (90)	5 (10)	50 (100)
	***E2* Gene Disruption**	***E2* Intact**	**Total**
	***n* (%)**	***n* (%)**	***n* (%)**
Normal	7 (70)	3 (30)	10 (100)
LSIL	19 (95)	1 (5)	20 (100)
HSIL	20 (100)	0 (0)	20 (100)
Total	46 (92)	4 (8)	50 (100)

## Data Availability

Data is contained within the article.
